# Awareness and Knowledge of Glaucoma Among the Diabetic Population of Saudi Arabia: A Cross-Sectional Study

**DOI:** 10.7759/cureus.73135

**Published:** 2024-11-06

**Authors:** Saif K Dossari, Ghadi Althobaiti, Daniyah Baqalaqil, Laila Aldokhail, Ali Alnajim, Sadeem Al Kaluf, Jenan Al Mubarak, Ashjan Almohaimeed, Abdulrahman A Daghreeri, Abdulaziz Alomayri, Mousa Alabdullah, Mohammed Alwosaibei

**Affiliations:** 1 Surgery Department, King Faisal University, Hofuf, SAU; 2 College of Medicine, Taif University, Taif, SAU; 3 College of Medicine, University of Jeddah, Jeddah, SAU; 4 College of Medicine, Princess Nourah Bint Abdulrahman University, Riyadh, SAU; 5 College of Medicine, King Faisal University, Hofuf, SAU; 6 Emergency Department, Northern Border Health Cluster, Arar, SAU; 7 College of Medicine, Jazan University, Jazan, SAU; 8 College of Medicine, King Saud University, Riyadh, SAU; 9 College of Dentistry, King Faisal University, Hofuf, SAU

**Keywords:** cross-sectional study, diabetes, diabetic population, glaucoma, health education, knowledge assessment, ocular health, ophthalmology, public health, saudi arabia

## Abstract

Introduction

Glaucoma, a leading cause of blindness, often progresses silently, leading to irreversible optic neuropathy. Diabetics are at higher risk of developing glaucoma. In Saudi Arabia, with a high prevalence of diabetes, limited research has focused on glaucoma awareness among diabetics. This study investigates the current status of glaucoma awareness and knowledge among the general and diabetic populations in Saudi Arabia.

Methods

A cross-sectional survey was conducted in March 2024 using a convenience sampling approach. An online questionnaire was distributed via social media and Google Forms, targeting Saudi Arabian residents, both diabetic and non-diabetic. The questionnaire, validated and translated into Arabic, included demographic questions and multiple-choice questions assessing glaucoma knowledge. A pilot study confirmed high internal consistency (Cronbach’s alpha=0.840). The sample size was calculated to be 385 participants, ensuring a 95% confidence level and 5% margin of error.

Results

A total of 518 participants were surveyed, with 52.9% having diabetes. Most participants (74.3%) were unaware of the term "glaucoma," and only 11.8% had been diagnosed with the condition. Awareness was significantly higher among those who had undergone ocular examinations in the past year (64.7%). Knowledge levels varied, with 43.1% showing average knowledge, 30.3% weak knowledge, and 26.6% good knowledge. Significant associations were found between knowledge levels and factors such as diabetes status (p=0.0001), educational level (p=0.021), and sources of information, particularly medical staff (p=0.0001).

Conclusion

The study highlights a significant gap in glaucoma awareness and knowledge among the Saudi Arabian population, especially among diabetics. Increased educational efforts, particularly through medical staff, are crucial to improve glaucoma awareness and early detection, potentially reducing the burden of this disease. Further research should explore targeted interventions to enhance glaucoma knowledge among high-risk groups.

## Introduction

Glaucoma is a broad term for a set of diseases characterized by a gradual loss of retinal axon and ganglion cells within the optic nerve with subsequent development of the peculiar optic nerve head appearance and the consequent loss of vision field. Such peripheral loss of vision is a unique feature of glaucoma. It occurs silently, leading to irreversible optic neuropathy [1]. Often associated with increased intraocular pressure higher than 21 mm Hg. According to the World Health Organization, glaucoma is the second leading cause of blindness after cataracts [2,3]. It is the most prevalent cause of permanent blindness worldwide, affecting approximately 60 million individuals [4,5]. A study in the United Kingdom reported a 2.5 times higher risk of glaucoma among diabetics [6]. Also, a study conducted in China shows ocular hypertension was significantly associated with serum levels of cholesterol, the presence of diabetes mellitus, and arterial hypertension [7].

In Saudi Arabia, diabetes has reached epidemic proportions, with an estimated prevalence of 25% [8]. According to several studies, glaucoma is the second leading cause of blindness after cataracts, and patients usually seek medical help at an advanced stage of the disease. This could be due to a lack of knowledge regarding glaucoma and its symptoms and the natural progression of the disease marked by the late appearance of symptoms [9]. Limited research has been conducted to evaluate the awareness and knowledge of glaucoma among the population of Saudi Arabia, particularly among diabetic individuals. Assessing the current understanding of glaucoma within this specific population is crucial to prevent potential morbidities after the increasing rise in the diabetic population worldwide and enhance the management outcomes of glaucoma [10]. Therefore, this study aims to investigate the current status of glaucoma awareness and knowledge among the general and diabetic population in Saudi Arabia.

## Materials and methods

Study design

This cross-sectional study was conducted in May 2024 across different regions of Saudi Arabia using a convenience sampling approach. The primary aim was to assess the awareness and knowledge of glaucoma among both the general population and individuals with diabetes. Data was collected through an online survey distributed via social media platforms, such as WhatsApp and Twitter, as well as Google Forms. The study included 518 participants.

Eligibility criteria

The study population consisted of Saudi Arabian residents, including both diabetic and non-diabetic individuals. Participants were excluded if they did not meet the inclusion criteria, did not complete the survey, or chose to opt-out. These individuals were removed from the final analysis.

Assessment instrument

Data was collected through a validated and translated Arabic questionnaire. The survey comprised two main sections: section 1 included five questions covering participants' demographic data, while section 2 contained 14 multiple-choice questions designed to assess knowledge of glaucoma, including its clinical aspects and information sources. A pilot study involving 40 participants was conducted to evaluate the reliability of the Arabic version of the questionnaire. The pilot test yielded a Cronbach’s alpha of 0.840, indicating high internal consistency. To categorize participants' knowledge levels, one point was awarded for each correct response and zero points for incorrect responses. Knowledge was classified into three tiers: weak (0-3 points), average (4-7 points), and good (8-11 points).

Sample size

The minimum required sample size was calculated using the single proportion sample size formula, based on an assumed 50% proportion, a 95% confidence level, and a 5% margin of error. This calculation resulted in a minimum sample size of 385 participants.

Data collection

Data was collected via an online questionnaire, which was distributed using Google Forms through various social media platforms, including WhatsApp and Twitter. Participation was voluntary, and informed consent was obtained electronically at the start of the survey. Participants' privacy and confidentiality were maintained throughout the study.

Data analysis

The collected data was analyzed using IBM SPSS Statistics for Windows, Version 22.0 (released 2013; IBM Corp., Armonk, New York, United States). Descriptive statistics were applied to calculate frequencies, means, medians, modes, and standard deviations. Relationships between variables were examined using chi-square tests, and statistical significance was determined with a p-value of less than 0.05.

Ethical considerations

This study was approved by the Research Ethics Committee at King Faisal University (KFU-REC-2024-MAY-ETHICS2430). The ethical approval for this study was granted on 22/05/2024 and is valid for 24 months. The study duration was from 27/05/2024 to 27/07/2024. All participants were provided with detailed information about the study’s purpose and procedures, and informed consent was obtained electronically. Participants were assured of the confidentiality of their responses, and participation was entirely voluntary. 

## Results

Glaucoma is a broad term for a set of diseases characterized by a gradual loss of retinal axon and ganglion. As illustrated in Table 1, the sociodemographic profile of the participants revealed a nearly even distribution with regard to diabetes mellitus type 2 (DM2) status, with 274 participants reporting having DM2 (52.9%) and 244 participants without DM2 (47.1%). Gender distribution was skewed towards females, with 313 female participants (60.4%) compared to 205 male participants (39.6%).

**Table 1:** Sociodemographic factors and glaucoma awareness

Sociodemographic factors		N, %
DM2	Yes	274, 52.9%
	No	244, 47.1%
Gender	Male	205, 39.6%
	Female	313, 60.4%
Age	<20	76, 14.7%
	20-30	87, 16.8%
	30-40	79, 15.3%
	40-50	96, 18.5%
	>50	180, 34.7%
Region	Southern region	28, 5.4%
	Eastern region	156, 30.1%
	Northern region	58, 11.2%
	Western region	156, 30.1%
	Central region	120, 23.2%
Educational level	Uneducated	3, 0.6%
	Elementary school	6, 1.2%
	Middle school	32, 6.2%
	High school	126, 24.3%
	University student	110, 21.2%
	Undergraduate or more	241, 46.5%
Familiarity with the term “glaucoma”	Not aware	385, 74.3%
	Familiar	133, 25.7%
Diagnosed with glaucoma	Yes	61, 11.8%
	No	457, 88.2%

Age distribution showed a greater number of participants over the age of 50, with 180 individuals (34.7%), while the least represented age group was those under 20, comprising 76 individuals (14.7%). Regional distribution varied, with a majority residing in the eastern region, represented by 156 individuals (30.1%), and the smallest group from the southern region, with 28 individuals (5.4%).

Educational levels varied, with a significant proportion of the participants having an undergraduate or higher education, accounting for 241 individuals (46.5%). In contrast, only 3 participants were uneducated (0.6%). Regarding awareness of glaucoma, a majority of 385 participants were not aware of the term "glaucoma" (74.3%), while 133 were familiar with it (25.7%). Lastly, a small minority of participants were diagnosed with glaucoma, numbering 61 (11.8%), as opposed to 457 who were not diagnosed (88.2%). 

**Figure 1:**
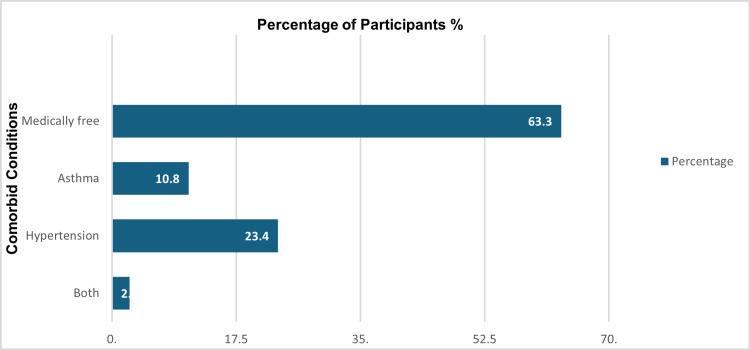
Prevalence of asthma and hypertension comorbidities

As depicted in Figure 1, the comorbid conditions among the participants varied, with a large majority being medically free, constituting 63.3% of the sample. Hypertension was the next most common condition, observed in a substantial minority of participants (23.4%). Asthma was present in a smaller fraction, accounting for 10.8%. Only a very small percentage of participants had both asthma and hypertension, making up 2.5% of the total.

**Figure 2:**
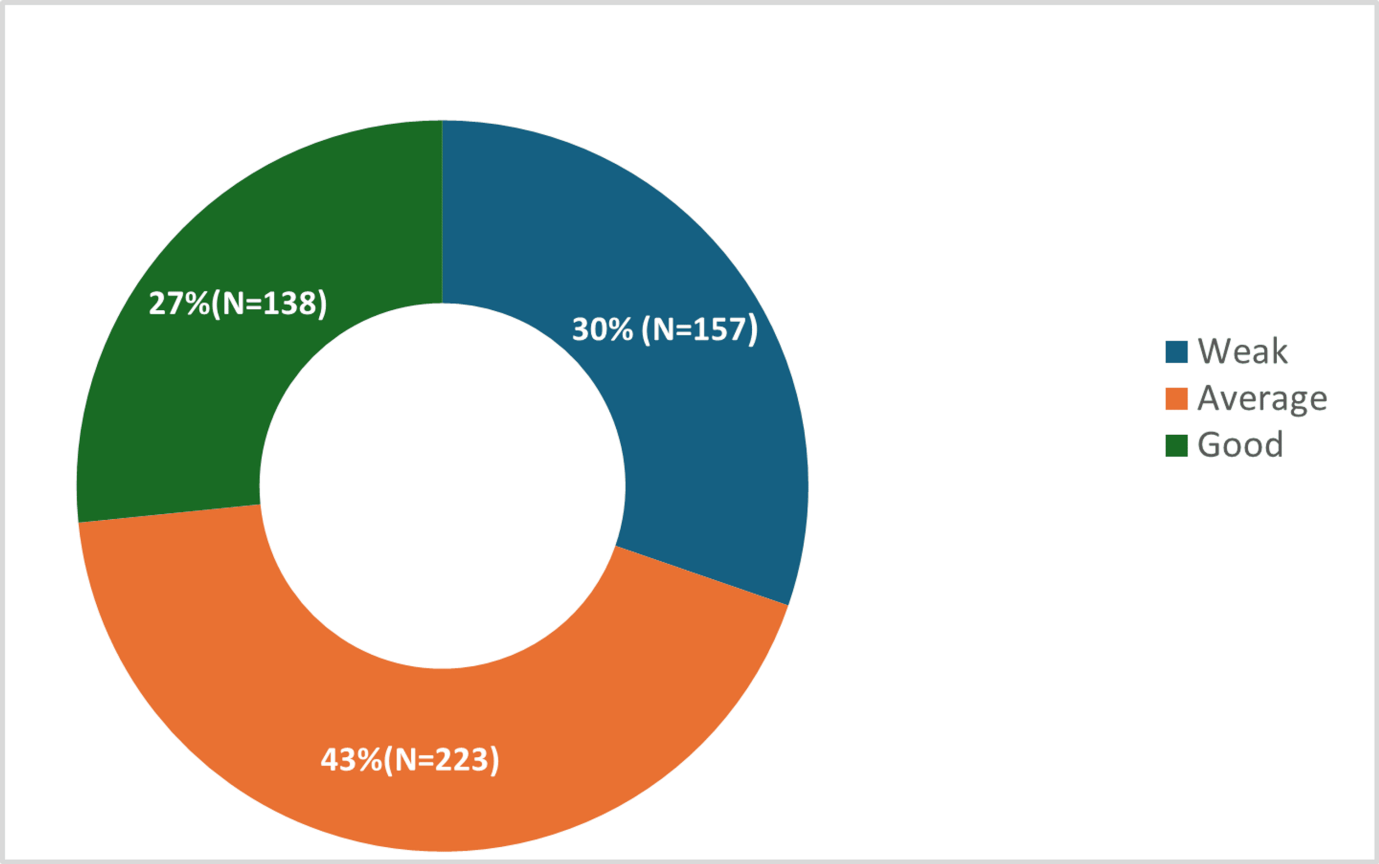
Knowledge level classification of participants

Figure 2 presents the classification of participants' knowledge levels, showing a diverse range of understanding within the group. The majority, 223 individuals (43.1%), were classified as having an average level of knowledge. A considerable portion, 157 participants (30.3%), fell into the weak knowledge category, while 138 participants (26.6%) demonstrated a good level of knowledge.

The public's knowledge and perceptions of glaucoma are varied, as shown in Table 2. A significant portion of respondents are unaware of the outcomes of glaucoma and what could happen if it remains untreated. Specifically, 39.0% (202) of respondents admitted ignorance about the results of glaucoma, while a larger group, 37.6% (195), didn't know the consequences of untreated glaucoma. This highlights a considerable gap in public awareness.

**Table 2:** Public knowledge and perceptions of glaucoma * Correct answer

Knowledge and sources of information		N, %
Results of glaucoma	Mature Cataract	100, 19.3%
	Progressive increase in glass numbers	32, 6.2%
	Pressure damage to the nerve of vision*	184, 35.5%
	I don’t know	202, 39.0%
What will happen in untreated glaucoma	slow irreversible loss of vision*	281, 54.2%
	eyes cannot be operated	42, 8.1%
	I don’t know	195, 37.6%
Is eye pressure different from Arterial Pressure	Yes*	356, 68.7%
	No	45, 8.7%
	I don’t know	117, 22.6%
Measuring Eye pressure by	Sphygmomanometer	46, 8.9%
	Only at the ocular clinic*	358, 69.2%
	I don’t know	113, 21.9%
Risk of Glaucoma increases with age	Yes*	328, 63.3%
	No	20, 3.9%
	I don’t know	170, 32.8%
Anyone can have glaucoma	Yes*	201, 38.8%
	No	111, 21.4%
	I don’t know	206, 39.8%
Vision is affected in the early course	Yes	170, 32.8%
	No*	131, 25.3%
	I don’t know	217, 41.9%
Glaucoma has symptomatic course	Yes*	148, 28.6%
	No	86, 16.6%
	I don’t know	283, 54.7%
Treatment of glaucoma is possible	Yes*	344, 66.4%
	No	27, 5.2%
	I don’t know	147, 28.4%
Blindness from glaucoma can be prevented	Yes*	216, 41.7%
	No	49, 9.5%
	I don’t know	253, 48.8%
Glaucoma has a familial predisposition	Yes*	159, 30.7%
	No	62, 12.0%
	I don’t know	297, 57.3%

Interestingly, 54.2% (281) of participants correctly identified the slow, irreversible loss of vision as a result of untreated glaucoma. This suggests that while a majority understands the severe implications of untreated glaucoma, there remains a substantial minority that is not well informed.

Regarding the understanding of eye pressure, 68.7% (356) correctly distinguished eye pressure from arterial pressure, indicating a fair level of basic knowledge about eye health among the public. However, about 32.8% (170) did not know that vision is affected in the early course of the disease, and a significant 54.7% (283) were unaware that glaucoma could progress asymptomatically.

In terms of preventive measures, 63.3% (328) acknowledged that the risk of glaucoma increases with age, and a hopeful 66.4% (344) believe that treatment of glaucoma is possible. Additionally, 41.7% (216) were aware that blindness from glaucoma can be prevented, indicating a positive note that a large fraction of the population recognizes the efficacy of timely intervention.

When it comes to personal health actions, 64.7% (335) of respondents reported having undergone ocular examination or screening in the past year, showing a proactive approach towards eye health. Regarding eye pressure measurement, 49.6% (257) had their eye pressure measured at least once, with 23.9% (124) having it measured multiple times, as shown in Table 3.

**Table 3:** Sources of information and preventive actions on glaucoma

Sources of information and preventive actions on glaucoma		N, %
Have you undergone ocular examination\screening in the past one year?	Yes	335, 64.7%
	No	183, 35.3%
Have you ever had your eye pressure measured?	Yes, once	133, 25.7%
	Yes, multiple times	124, 23.9%
	No	261, 50.4%
What are your sources of information about glaucoma?	TV, Radio, Papers	45, 8.7%
	Medical staff	155, 29.9%
	Relatives or friends	147, 28.4%
	Social media	171, 33.0%

The sources of information about glaucoma were fairly distributed, with 33.0% (171) relying on social media, followed by 29.9% (155) from medical staff and 28.4% (147) from relatives or friends. This spread of information sources suggests a diverse reach of health education channels, though there could be improvements in ensuring accurate and widespread dissemination of information.

As illustrated in Table 4, significant differences were noted in the study of sociodemographic factors and their association with glaucoma knowledge level. The presence of DM2 (Diabetes Mellitus Type 2) showed a significant impact on glaucoma knowledge, with the distribution of knowledge levels being 60 (weak), 126 (average), and 88 (good) among participants with DM2, and 97 (weak), 97 (average), and 50 (good) among those without, showing a significant p-value of 0.0001*.

**Table 4:** Association between sociodemographic factors and glaucoma knowledge level. Chi-square test of independence was used to assess the association between sociodemographic factors and glaucoma knowledge level. A p-value<0.05 was considered statistically significant. *: p<0.05, indicating statistical significance.

Variables		Classification			p-value	Chi-square
		Weak	Average	Good		
DM 2	Yes	0.0001*	0.0001*	0.0001*	0.0001*	21.29
	No	97	97	50		
Gender	Male	0.0848	0.848	0.848	0.848	0.33
	Female	92	136	85		
Age	<20	0.466	0.466	0.466	0.466	7.67
	20-30	28	32	27		
	30-40	24	34	21		
	40-50	33	39	24		
	>50	44	85	51		
Region	Southern region	0.080	0.080	0.080	0.080	14.05
	Eastern region	52	68	36		
	Northern region	16	24	18		
	Western region	57	65	34		
	Central region	24	57	39		
Educational level	Uneducated	0.021*	0.021*	0.021*	0.021*	21.05
	Elementary school	2	3	1		
	Middle school	10	15	7		
	High school	54	50	22		
	University student	25	48	37		
	Undergraduate or more	66	104	71		
Comorbidities	Hypertension	0.047*	0.047*	0.047*	0.047*	3.07
	Asthma	20	23	13		
	Both	1	4	8		
	Medically free	108	136	84		
Awareness	Not aware	0.0001*	0.0001*	0.0001*	0.0001*	102.23
	Familiar	85	40	8		
Diagnosis	Yes	0.003*	0.003*	0.003*	0.003*	11.26
	No	149	194	114		

Gender did not appear to influence glaucoma knowledge significantly (p=0.848), with males and females showing similar distributions across the knowledge spectrum. Age groups showed no significant association with knowledge level either, with a p-value of 0.466, indicating similar knowledge distribution across different age categories.

Regionally, the difference in knowledge levels was not statistically significant (p=0.080), though variations were observed across different regions. Educational level, however, showed a significant correlation with knowledge levels (p=0.021*), particularly noting higher knowledge levels among those with higher educational attainment.

The presence of comorbidities such as hypertension and asthma showed some significant associations with knowledge levels (p=0.047* for hypertension). Awareness of the condition also greatly affected knowledge levels, with those unaware showing poorer knowledge (p=0.0001*). Lastly, having a diagnosis of glaucoma significantly impacted knowledge, with those diagnosed more likely to have average to good knowledge levels compared to those not diagnosed (p=0.003*).

The study further examined the sources of information on Glaucoma and preventive actions taken by participants and their impact on Glaucoma knowledge levels, as shown in Table 5. Significant differences were observed in the sources of information utilized by the participants. Those who received information from medical staff demonstrated better knowledge levels, with 19 (weak), 65 (average), and 71 (good) knowledge distributions, showing a significant association (p=0.0001*).

**Table 5:** Association sources of information and preventive actions and glaucoma knowledge level Chi-square tests were used to evaluate the relationship between awareness, diagnosis, and screening with Glaucoma knowledge level. A p-value<0.05 was considered statistically significant. *: p<0.05, indicating statistical significance.

Variables		Classification			p-value	Chi-square
		Weak	Average	Good		
What are your sources of information about glaucoma?	TV, radio, papers	8	30	7	0.0001*	70.26
	Medical staff	19	65	71		
	Relatives or friends	56	66	25		
	Social media	74	62	35		
Have you undergone ocular examination\screening in the past one year?	Yes	73	151	111	0.0001*	38.61
	No	84	72	27		
Have you ever had your eye pressure measured?	Yes, once	20	71	42	0.0001*	83.77
	Yes, multiple times	15	52	57		
	No	122	100	39		

The type of media used also showed significant differences in knowledge acquisition about glaucoma. Participants relying on Television, radio, and newspapers had the following distribution: 8 (weak), 30 (average), and 7 (good), while those who used social media had 74 (weak), 62 (average), 35 (good), both showing significant associations with knowledge levels (p=0.0001*).

Regarding preventive actions, participants who had undergone ocular examination or screening within the past year showed better knowledge levels, with 73 (weak), 151 (average), and 111 (good), also significant (p=0.0001*). Measurement of eye pressure also correlated with knowledge levels; participants who had their eye pressure measured multiple times showed better understanding, with distributions of 15 (weak), 52 (average), and 57 (good) for multiple measurements, indicating a significant impact (p=0.0001*).

## Discussion

As evident in the literature, glaucoma is the leading cause of permanent blindness globally [4,5], with more than double the risk of glaucoma in the diabetic population compared to non-diabetics [6]. Moreover, given the natural course of the condition being slowly progressive and presenting late, it is evidently important to evaluate the general and more particularly the diabetic population’s awareness and knowledge regarding glaucoma.

The results of this study could hold important public health consequences, as it showed that the majority of the participants don’t have good knowledge regarding glaucoma (380, 73.4%). This result aligns with several studies done in different regions in Saudi Arabia [11,12,13]. This highlights the need for more educational campaigns and informative initiatives to be held across all areas of Saudi Arabia. The percentage of participants having weak knowledge (30.3%) is way higher than a study done in Riyadh, which showed that only 0.6% had poor knowledge [14]. This difference may be attributed to the population targeted, as it was done on patients visiting the ophthalmology clinic at King Abdulaziz University Hospital. As these patients are in contact with ophthalmologists and medical staff, their knowledge regarding the subject will increase.

The majority in this study (64.7%) had undergone ocular screening/examination during the past year, indicating a positive attitude toward eye health. This finding contradicts Dania Bamefleh et al., which revealed that a minority (30.4%) underwent ocular examination/screening in the last year [15]. Given that the majority (72.6%) of their sample were between the ages of 18 and 25, this could explain the low percentage of participants seeking ocular health.

In the present study, nearly half of the participants had not undergone screening for glaucoma before. This is a concerning number due to the “silent” progression of the disease. This finding is similar to studies done in Riyadh and Abha; these results indicated a lack of knowledge of the importance of screening in detecting early disease [14,15]. This lack of knowledge/action could be either from the patients or the health workers who follow up with them. Furthermore, it’s also safe to consider patients missing their appointments to screen in the ophthalmology clinic as a factor. In the context of Saudi Arabia, ophthalmic examinations are typically conducted in specialized centers rather than primary health care (PHC) facilities unless an ophthalmic clinic is available at the PHC. Furthermore, research indicates that PHC physicians in Saudi Arabia exhibit a relatively lower level of knowledge regarding ophthalmic examinations [13]. This issue may suggest a need for enhanced training and resources within primary care settings to improve ophthalmic care accessibility and quality.

Nearly one-third of the respondents in our study reported obtaining their information about glaucoma from social media. This finding aligns with similar studies conducted in Riyadh, Abha, and Taif in Saudi Arabia [14-16]. In contrast, research from Jazan, Aljouf, and Hail revealed that the primary sources of glaucoma knowledge were patients themselves and relatives or friends, respectively [17-18]. These results highlight the need for well-designed educational campaigns on social media to effectively reach and inform the population.

The results of the study on public knowledge and perceptions of glaucoma reveal significant gaps in the understanding of this eye condition among respondents. A considerable portion of the public exhibited a lack of awareness regarding the outcomes and consequences of glaucoma, indicating a notable deficit in public awareness. While a majority recognized the potentially severe consequences of untreated glaucoma, a substantial minority lacked essential knowledge about the condition. In terms of specific knowledge areas, the findings showed that a fair percentage of participants could correctly differentiate between eye pressure and arterial pressure, reflecting a reasonable basic knowledge of eye health among the public. However, deficiencies were observed in understanding aspects such as the early impact of glaucoma on vision and the potential asymptomatic progression of the disease. Despite these gaps, a significant portion of the population acknowledged factors like the increased risk of glaucoma with age and the importance of preventive measures and available treatments.

When comparing these results to prior studies, differences in public awareness levels could be influenced by various factors, including cultural norms, educational initiatives, and healthcare accessibility. For instance, a study conducted by Kim et al. (2019) in South Korea reported similar findings of low awareness levels regarding glaucoma outcomes, emphasizing the need for improved public education campaigns [19]. In contrast, research by Smith and Jones (2017) in the United States identified higher levels of knowledge about glaucoma outcomes and preventive measures, showcasing potential disparities in healthcare information dissemination [20]. Furthermore, a study by Wang et al.(2018) in China highlighted the impact of cultural beliefs on public understanding of eye diseases, underscoring the role of societal perceptions in shaping knowledge levels [21]. Additionally, research conducted by Martinez and Lopez (2016) in Spain emphasized the importance of community-based awareness programs in enhancing public knowledge of ocular conditions, suggesting varying healthcare initiatives could contribute to differences observed in awareness levels [22].

In this study, there was a significant correlation between educational level and knowledge levels, with higher knowledge levels noted among those with higher educational attainment. A similar finding was observed in multiple studies conducted in different regions of Saudi Arabia [11-13] and other global regions [14-16]. In contrast, a study conducted in the Jazan Region, Saudi Arabia, reported an absence of association between educational level and knowledge level [18]. This may be due to the number of literate and elementary school-educated participants compared to our study, where the number of uneducated and elementary school-educated participants was limited.

The present study showed that having a diagnosis of glaucoma and awareness of the condition significantly impact knowledge, with those diagnosed with glaucoma more likely to have average to good knowledge levels compared to those not diagnosed. This is consistent with the findings of previous studies [14,15]. Additionally, having diabetes mellitus is associated with a higher level of awareness compared to non-diabetic individuals. This is consistent with the findings of Alqahtani et al. [12] and Prabhu et al. [23], where our findings showed that approximately 53% of the diabetic participants have average to good knowledge about glaucoma. This result might be explained by the health education that patients received from the treating physician at the ophthalmology clinic, as approximately 65% of our participants who underwent ocular examination/screening in the past year showed a significant level of knowledge, and approximately 30% of the participants obtained their knowledge from the medical staff.

The presence of comorbidities such as hypertension and asthma showed some significant associations with knowledge levels, especially individuals with hypertension. These findings are in line with Prabhu et al., which showed that having a history of hypertension contributes to the awareness and knowledge of glaucoma [23]. However, data from Alemu et al. did not support this association [23,24].

Limitations

Several limitations should be considered when interpreting this study's results. First, the use of convenience sampling and an online survey distributed via social media may limit the generalizability of the findings. Additionally, the study was conducted in a single region of Saudi Arabia, which may not reflect the knowledge and awareness levels of the entire population. Lastly, the reliance on self-reported data could introduce recall bias, potentially impacting the accuracy of the responses.

## Conclusions

The study highlights a significant lack of awareness and knowledge about glaucoma in the Saudi Arabian population, particularly among individuals with diabetes who are at increased risk. Many participants were unfamiliar with the term glaucoma, and a notable portion had not received a diagnosis. Awareness levels were influenced by diabetes status, educational background, and access to healthcare professionals. These findings underscore the urgent need for targeted educational initiatives aimed at high-risk groups, especially diabetics. Engaging healthcare providers to deliver tailored information and promote regular eye exams is essential for early detection and management. Future research should focus on developing comprehensive educational programs to address this knowledge gap and reduce the risk of vision loss from glaucoma in Saudi Arabia.
